# Lactylation-Related Biomarkers in Heart Failure with Reduced Ejection Fraction: Association of Serum HMGN2 with Prevalent Heart Failure and Its Diagnostic Performance

**DOI:** 10.3390/diagnostics16213665

**Published:** 2026-11-05

**Authors:** Zongzhuhai Yu, Wanwan Chen, Yuanyi Wu, Jianfei Wang, Liangjie Xu

**Affiliations:** 1 Department of Cardiology, Affiliated People’s Hospital of Jiangsu University, Zhenjiang 212001, China; 2 Department of Cardiology, Affiliated Hospital of Jiangsu University, Zhenjiang 212050, China

**Keywords:** lactylation, high-mobility group nucleosomal-binding domain 2, heart failure with reduced ejection fraction, prognostic value

## Abstract

**Background/Objectives:** Lactate and lactylation are associated with the development of heart failure with reduced ejection fraction (HFrEF). This study aimed to identify potential lactylation-related biomarkers with diagnostic and prognostic value in patients with HFrEF. **Methods**: We identified the potential lactylation-related biomarkers for heart failure (HF) by integrating bioinformatics analysis with machine learning algorithms. A total of 251 patients with HFrEF and 210 adults without HF were enrolled to validate the diagnostic values of lactylation-related biomarkers at both the mRNA and protein levels. **Results**: Bioinformatics analysis and machine learning identified *EIF4G1*, *HLTF*, *HMGN2*, *LSP1*, and *PPM1G* as hub lactylation-related genes in patients with HF. In the external validation cohort, elevated serum high-mobility group nucleosomal-binding domain 2 (HMGN2) levels were significantly associated with HFrEF. Beyond its diagnostic utility in HFrEF, HMGN2 was associated with prevalent HFrEF (AUC 0.81, 95% CI 0.77–0.85). In exploratory 12-month follow-up, higher HMGN2 was associated with poor outcomes. **Conclusions**: HMGN2 is a lactylation-associated biomarker that is elevated in patients with HFrEF, associated with prevalent HFrEF, and shows promise for risk stratification with diagnostic discrimination.

## 1. Introduction

Heart failure (HF) severely impairs patients’ quality of life and increases their risks of all-cause and cardiovascular mortality, and it is currently recognized as a global pandemic [1]. Despite substantial progress in pharmacological therapies over the past several decades, the prognosis of HF remains dismal. Thus, the early detection of optimal biomarkers and control of associated risk factors are important for preventing and slowing HF progression. The heterogeneous pathophysiology of HF, including metabolic reprogramming, oxidative stress, and adverse cardiac remodeling, underscores the complexity of developing effective prognostic biomarkers [2]. In particular, growing evidence indicates that metabolic disorders, especially altered glucose metabolism and lactate homeostasis, play a pivotal role in the initiation and progression of HF. Lactate is not only regarded as a metabolic waste product, but it is also involved in organ development, acts as a precursor for gluconeogenesis [3], enters the mitochondrial matrix to provide energy [4], modulates immunity, maintains the homeostasis of subcellular organelles [5,6] and controls cell proliferation [7]. Furthermore, lactate can serve as an epigenetic modification substrate, causing histone or nonhistone lysine residues to undergo lactylation, which then regulates gene transcription or protein function [8,9,10,11]. Many studies have shown that high levels of lactate are associated with poor prognosis in patients with acute HF [12]. With the deepening of research in recent years, emerging evidence underscores the critical role that lactylation plays in HF progression. Lactate regulates pathological cardiac hypertrophy [13], drives neutrophil extracellular trap formation [14], and activates the expression of proline hydroxylase and TGF-β1, as well as the hydroxylation of collagen [15], via lactylation modification. Recently, Yingxian Sun’s team found that the downregulation of α-myosin heavy chain K1897 lactylation impairs the α-MHC-Titin interaction and worsens HF [16]. These cumulative observations demonstrate that lactylation serves as a link between metabolic perturbation and HF [17].

Advances in bioinformatics and database analyses have validated that lactylation contributes to the development of HF. JAK/STAT signaling pathway-related genes exhibited positive correlations with M1 macrophage infiltration, which establishes a mechanistic connection between lactylation and excessive inflammatory response in HF [18]. HSPA2, BRD4, MYH6, HBB, EXT1, CENPA, NT5E, STAT4, and CAPN5 levels were significantly higher in patients with HF [19,20]. However, a common limitation the above investigations shared is the absence of survival information or external validation of their prognostic signatures.

Accordingly, the present study was designed to address two specific gaps left by these earlier investigations. First, previous lactylation-associated signatures for heart failure were constructed and validated only at the mRNA level; we extended validation to the circulating protein level in an independent clinical cohort. Second, previous studies were confined to cross-sectional diagnostic discrimination and lacked longitudinal outcome follow-up. In our study, all patients underwent 12 months of prospective follow-up based on a prespecified composite endpoint. Using integrated bioinformatics and machine learning, we aimed to establish a lactylation-associated gene signature for prognostic prediction in patients with heart failure with reduced ejection fraction (HFrEF), followed by validation in an external cohort. This work provides further insights into the association between lactylation and HFrEF, and the potential role of HMGN2 as a candidate prognostic biomarker in patients with HFrEF.

## 2. Materials and Methods

### 2.1. Data Sources

Bulk RNA sequencing datasets were downloaded from the GEO database: GSE57345 (https://www.ncbi.nlm.nih.gov/geo/acc.cgi?acc=GSE57345) (accessed on 15 March 2025), comprising RNA expression profiles of 313 individuals with or without abnormal left ventricular ejection fraction (LVEF), and GSE5406 (https://www.ncbi.nlm.nih.gov/geo/acc.cgi?acc=GSE5406) (accessed on 15 March 2025), comprising RNA expression profiles of 210 individuals with or without abnormal LVEF. The training dataset was derived from GSE57345, and the validation dataset was derived from GSE5406.

### 2.2. Differential Expression Analysis

A total of 371 lactylation-related genes (LRGs) were obtained from previously reported data (PMID: 37242427) and Gene Ontology (http://geneontology.org//) (Table S1). The original dataset was categorized as follows: “Non_failing_Left_Ventricle” was set as the control group, whereas “Idiopathic Dilated CMP and Ischemic” was set as the HF group. We used the limma R package (3.64.x) to calculate *p*-values and log fold changes (logFC) in gene expression. Benjamini–Hochberg correction was performed to reduce the bias caused by multiple tests. Differentially expressed genes (DEGs) were identified using an absolute log2 fold change ≥2 and an adjusted *p*-value < 0.05. To identify lactylation-related DEGs in patients with HF, we merged the 371 LRGs with the DEGs identified in patients with HF.

### 2.3. Machine-Learning-Based Identification of Optimal Feature Genes

In the GSE57345 dataset, the number of LRGs was large; therefore, we screened the hub lactylation-related DEGs using machine learning to identify the optimal feature genes. Three machine-learning algorithms were used for hub gene identification: least absolute shrinkage and selection operator (LASSO) [21], support vector machine-recursive feature elimination (SVM-RFE), and random forest (RF). We used LASSO regression with 10-fold cross-validation to select the optimization parameters that satisfied the minimum criteria. The “glmnet” package in R was used to construct an optimal LASSO logistic regression model. For SVM-RFE, the rfeControl function was configured with the parameters method = “cv” and number = 10 [22]. For the RF algorithm, the R package “randomForest” was used to explore the optimal value. Genes identified by all three algorithms (LASSO, SVM-RFE, and RF) were defined as hub lactylation-related DEGs.

#### Expression and Predictive Significance of Optimal Feature Genes in HF Tissues

The “stats” package (https://stat.ethz.ch/R-manual/R-devel/library/stats/html/00Index.html) (accessed on 15 March 2025), in R was used to assess the diagnostic performance of the hub lactylation-related DEGs. Receiver operating characteristic (ROC) curves were generated for these genes in both the training and validation datasets, and the corresponding area under the curve (AUC) values were calculated.

### 2.4. Enrichment Analysis

To identify specific biological pathways enriched in the lactylation-related DEGs, we used the “clusterProfiler” package to perform GO and KEGG functional enrichment analyses to elucidate the functional roles of the screened candidate genes and their associated pathways [5].

### 2.5. An External Validation Cohort

**Figure 1:**
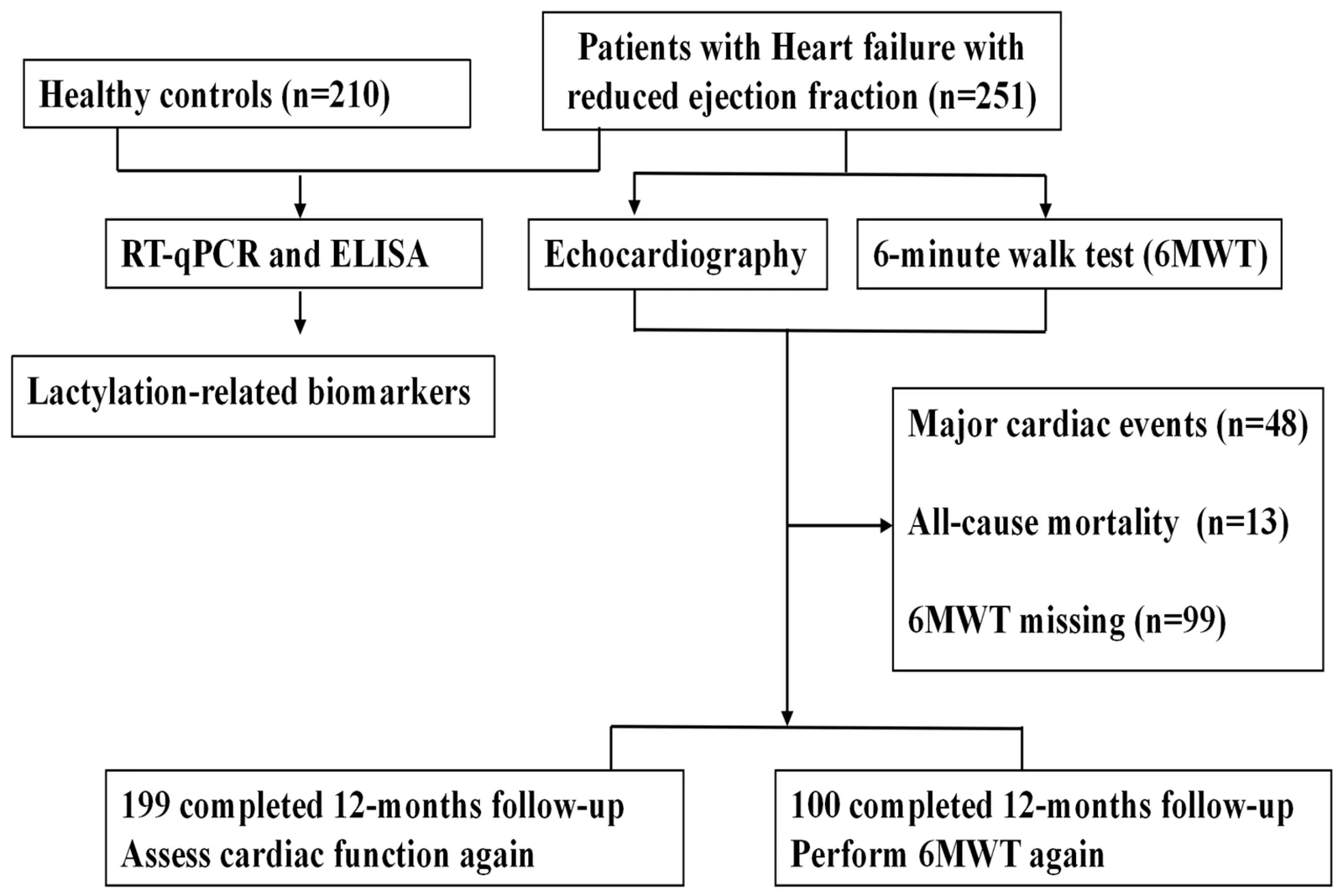
Flow chart showing the enrollment protocol.

To validate the findings from the bioinformatics analyses, a total of 461 participants were enrolled in this study, comprising 251 patients with HFrEF and 210 age- and sex-matched healthy controls (Figure 1). Eligible patients with HFrEF met the following inclusion criteria [23,24]: (1) presence of typical HF-related symptoms and physical signs; (2) LVEF ≤ 49%; and (3) age above 18 years. Patients with known liver disease, those on dialysis, those receiving anemia treatment, or those with a clinical diagnosis of cardiogenic shock were excluded from the study. This study was approved by the Ethics Committee of the Affiliated People’s Hospital of Jiangsu University (Approval No. K20250074Y).

The 6-min walk test (6MWT) was performed in a long, straight hospital corridor, over a 30 m distance [25]. A ≥15 m improvement was chosen as a clinically meaningful threshold [26]. Improvement in LVEF was defined as an absolute increase of more than 10% from baseline at the 12-month follow-up. Patients in the validation cohort were followed up at 2, 4, 6, 8, and 12 months. Follow-up assessments were conducted through clinical visits and telephone interviews to document the occurrence of the primary endpoint, which included all-cause mortality and major cardiac events [27]. Major cardiac events were defined as newly occurring myocardial ischemia, arrhythmia, and HF events. A new HF event was defined as HF rehospitalization, HF-related emergency department visit, or medication up-titration required because of worsening clinical status or signs/symptoms of HF decompensation.

### 2.6. Echocardiographic Measurement

Conventional echocardiographic parameters and left ventricular global longitudinal strain (GLS) were acquired using a Philips CVX system. GLS was used to assess early left ventricular systolic dysfunction and was presented as absolute values.

### 2.7. Real-Time Quantitative Polymerase Chain Reaction

We isolated peripheral blood mononuclear cells (PBMCs) from the patients with HF and healthy controls. RNA extraction and real-time quantitative polymerase chain reaction were performed following standard protocols. The primer sequences used are listed in Table S2.

### 2.8. Enzyme-Linked Immunosorbent Assay (ELISA)

Blood samples (5 mL each) were collected and centrifuged at 2000× *g* for 15 min at 4 °C within 2 h of collection [28]. The resulting serum was then aliquoted into 500 μL cryovials and immediately stored at −80 °C until analysis. High-mobility group nucleosomal-binding domain 2 (HMGN2) expression levels were quantified using a commercial biotin/avidin-based enzyme-linked immunosorbent assay kit.

### 2.9. Use of Generative AI Tools

Artificial intelligence was not used in the preparation of this manuscript.

### 2.10. Statistical Analyses

All gene expression data were normalized using log2 transformation. Descriptive statistics were reported as mean ± standard deviation (SD) or median ± interquartile range (IQR), depending on the data distribution. Continuous variables were analyzed using either analysis of variance (ANOVA) or the Mann–Whitney U test, as appropriate. Receiver operating characteristic (ROC) curve analysis was performed to determine sensitivity and specificity values using the Youden index. Bivariate correlations were assessed using Spearman’s rank correlation tests. The independent effect of HMGN2 on HF occurrence after controlling for baseline clinical characteristics and echocardiographic parameters (adopted factors: gender, BSA, BMI, heart rate, history of CAD, atrial fibrillation, diabetes mellitus, hyperlipidemia, smoking, SBP, renal dysfunction, creatinine, PCO_2_, HCO_3_^−^ , lactate, procollagen type I, procollagen type III, MMP-9, NT-proBNP, IL-6, and HMGN2) was assessed via logistic regression analysis. Cox proportional hazards regression models were used to evaluate the relationships between the HMGN2 levels and the primary endpoint. Statistical analyses were performed using SPSS software (version 19.0, Chicago, IL, USA) and GraphPad Prism (version 8.0, San Diego, CA, USA). A *p* value < 0.05 was considered statistically significant.

## 3. Results

### 3.1. Identification of LRGs in Patients with HF

**Figure 2:**
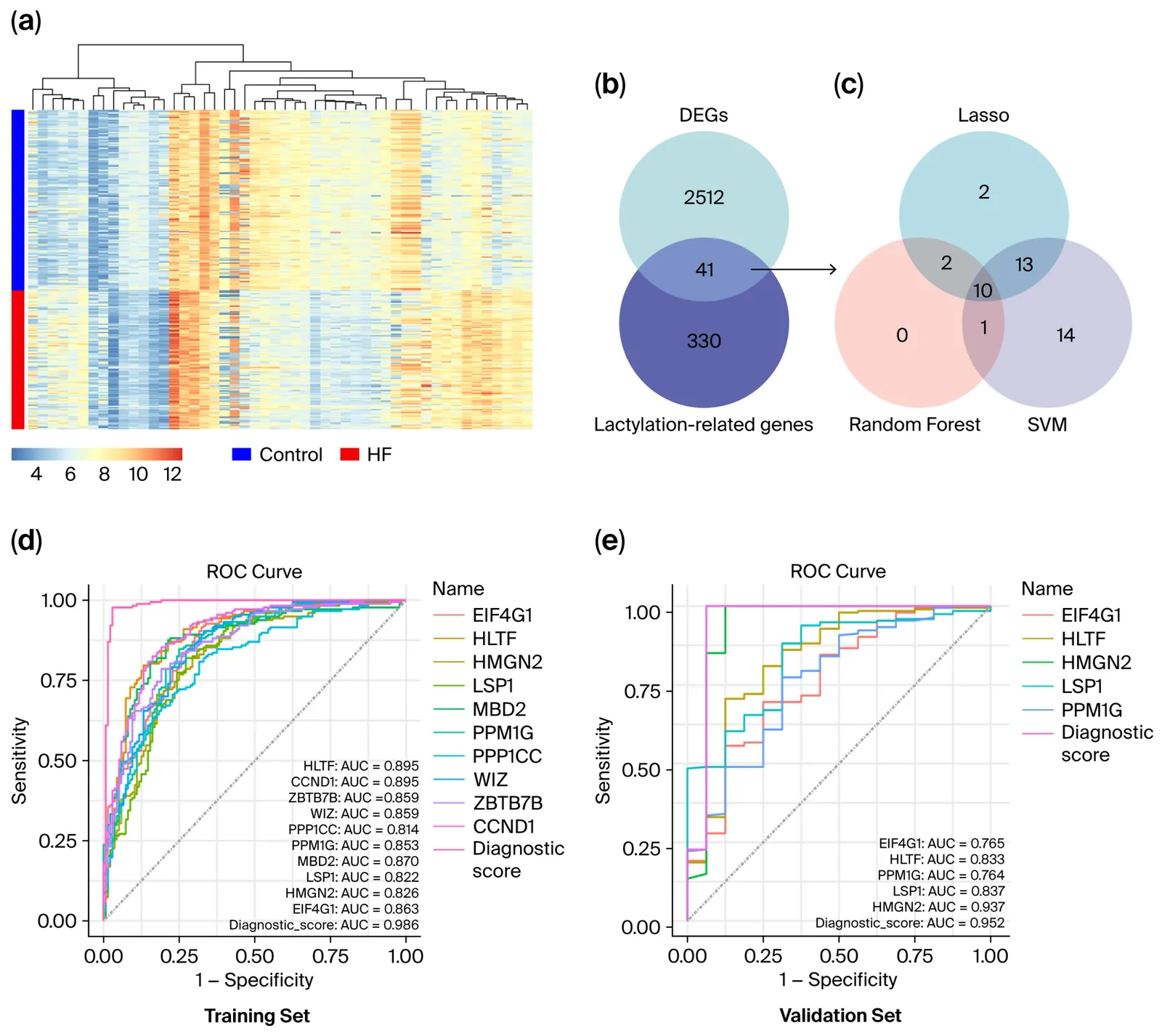
Identification of five hub genes of lactylation. (**a**) Heat map showing genes that differed between ejection fraction and control. (**b**) Wayne diagram showing the intersection of differentially expressed genes with lactylation genes. (**c**) Intersection of the results obtained by the above three methods (LASSO regression, random forest algorithm, and support vector machine) to obtain ten hub genes. (**d**) Receiver operating characteristic curves of ten genes (*EIF4G1*, *HLTF*, *HMGN2*, *LSP1*, *MBD2*, *PPM1G*, *PPP1CC*, *WIZ*, *ZBTB7B*, and *CCND1*) for predicting the occurrence of HF based on the training set (GSE57345). (**e**) Receiver operating characteristic curves of five genes (*EIF4G1*, *HLTF*, *HMGN2*, *LSP1*, and *PPM1G*) for predicting the occurrence of HF based on the validation set (GSE5406).

A total of 371 LRGs were obtained from previously reported data (PMID: 37242427) and Gene Ontology (http://geneontology.org//) (Table S1). In the GSE57345 dataset, we acquired expression profiles for 177 patients with HF and 136 healthy controls. There were 2553 DEGs, comprising 1142 upregulated genes and 1411 downregulated genes. These DEGs were visualized using volcano plots and heatmaps (Figure 2a and Figure S1A). To identify lactylation-related DEGs in patients with HF, we determined the overlap between the 371 LRGs and DEGs from GSE57345; 41 candidate genes were obtained as the lactylation-related DEGs in patients with HF (Figure 2b, Table S3). GO function and KEGG pathway analyses are shown in Figure S1C,D. GO analysis indicated significant enrichment in “lactylation”, “nucleoside diphosphate metabolic process”, “body fluid secretion”, “mammary gland development”, and “regulation of body fluid levels”. Of note, “mammary gland development” and “body fluid secretion” most likely reflect the limited size and compositional bias of the input gene list, are reported for completeness only, and are not interpreted mechanistically. KEGG analysis showed a strong correlation of HF with “glycolysis/gluconeogenesis”, “HIF-1 signaling pathway”, “central carbon metabolism in cancer”, “pentose phosphate pathway”, and “carbon metabolism”. Collectively, this series of analyses uncovered 41 lactylation-related genes that not only demonstrated significantly altered expression in HF but also exhibited a strong association with lactate-related metabolic pathways. We constructed a protein–protein interaction network of hub genes and visualized it using Cytoscape software (version 3.9.1; Cytoscape Consortium, San Diego, CA, USA) (Figure S1B). The previously screened lactylation-related hub genes are highlighted in orange, and seven hub genes (*EIF4G1*, *HMGN2*, *MBD2*, *PPM1G*, *PPP1CC*, *WIZ*, and *CCND1*) were obtained.

### 3.2. Identification of Hub LRGs in Patients with HF

To investigate the hub LRGs in HF, three machine-learning algorithms (namely, LASSO regression, the SVM-RFE algorithm, and RF analysis) were used to further filter feature genes. First, LASSO regression was performed on the 41 LRGs, yielding 27 candidate genes (Figure S2A,B). Subsequently, the SVM-RFE algorithm was adopted to rank these genes, and 38 hub genes were screened (Figure S2D). Meanwhile, random forest analysis identified 13 hub genes (Figure S2C). Finally, we intersected the gene sets derived from the three algorithms and identified 10 lactylation-related genes, namely *EIF4G1*, *HLTF*, *HMGN2*, *LSP1*, *MBD2*, *PPM1G*, *PPP1CC*, *WIZ*, *ZBTB7B*, and *CCND1*(Figure 2c). Subsequently, ROC curve analysis was performed to evaluate the diagnostic potential of these 10 hub LRGs for differentiating patients with HF from healthy controls. All 10 genes displayed favorable discriminatory performance in the training dataset, with AUC values exceeding 0.8 (Figure 2d). We downloaded the GSE5406 dataset as a validation dataset to further verify the diagnostic efficacy of the 10 lactylation-related hub genes. ROC curve analysis revealed that *EIF4G1*, *HLTF*, *HMGN2*, *LSP1*, and *PPM1G* possessed robust diagnostic performance for HF (Figure 2e). Accordingly, these five genes were ultimately defined as the hub LRGs for HF.

### 3.3. Functional Analysis of Hub LRGs in HF

*EIF4G1* expression exhibited a positive correlation with the VEGF signaling pathway and a negative correlation with antigen processing and presentation (Figure S3A). *HLTF* expression exhibited positive correlations with the VEGF signaling pathway, the B cell receptor signaling pathway, apoptosis, and the MAPK signaling pathway, and a negative correlation with antigen processing and presentation (Figure S3B). *HMGN2* expression showed a negative correlation with antigen processing and presentation (Figure S3C). *LSP1* expression showed a positive correlation with the VEGF signaling pathway and a negative correlation with antigen processing and presentation (Figure S3D). *PPM1G* expression showed a positive correlation with the VEGF signaling pathway and a negative correlation with antigen processing and presentation (Figure S3E).

### 3.4. Single-Cell Analysis of Hub LRGs in HF

**Figure 3:**
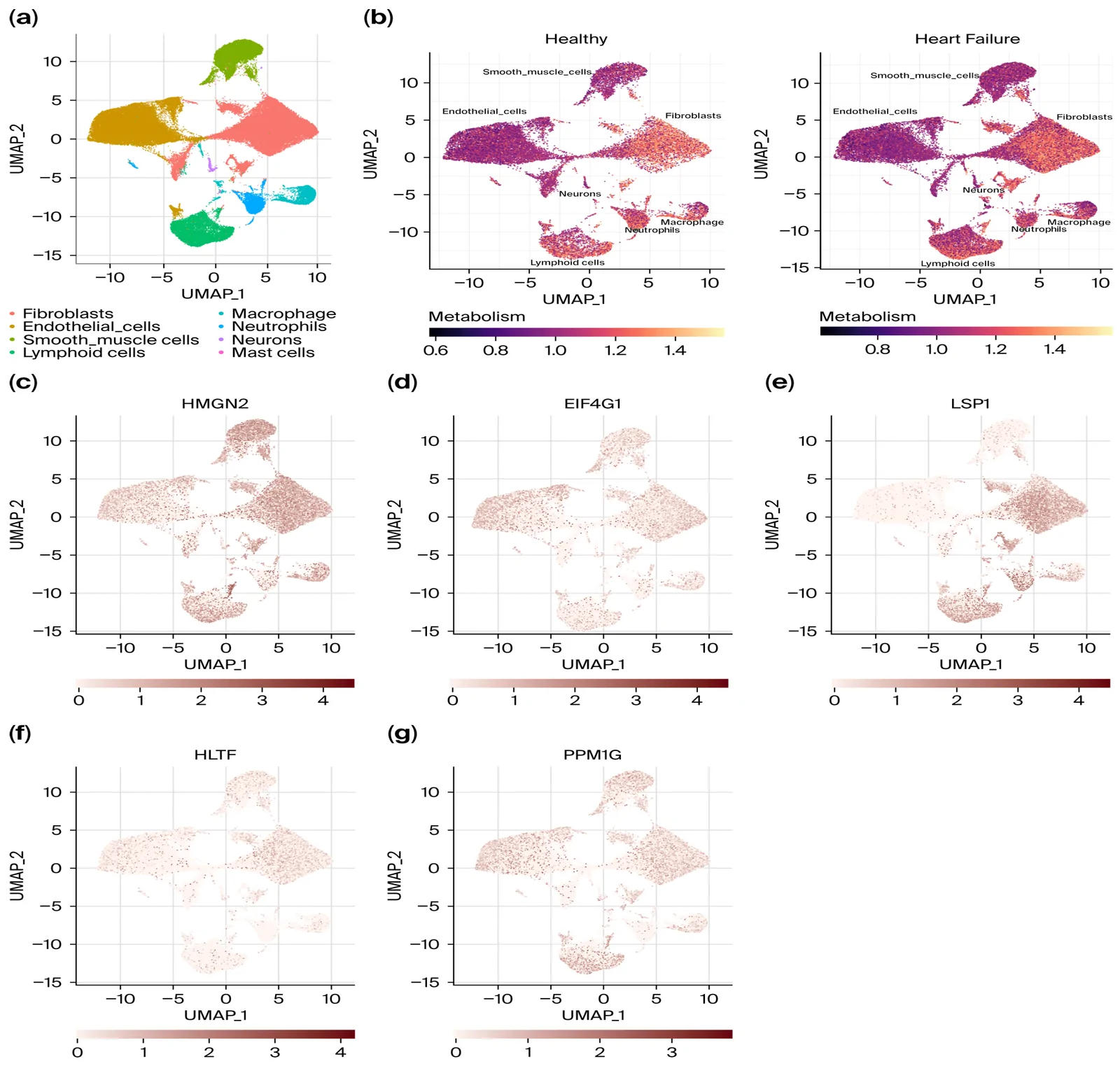
Single-cell RNA sequencing analysis. (**a**) Cluster annotation and cell type identification by means of UMAP. (**b**) UMAP plots of healthy controls and patients with heart failure. (**c**–**g**) Expression levels of five hub lactylation-related genes across single-cell data (HMGN2, EIF4G1, LSP1, HLTF, and PPM1G).

To characterize the cellular expression landscape of hub LRGs in HF, we downloaded single-cell RNA sequencing (scRNA-seq) data from the GSE247468 dataset, which contained samples from two healthy controls and four patients with HF. After using Uniform Manifold Approximation and Projection to reduce dimensions, eight cell clusters were identified: fibroblasts, endothelial cells, smooth muscle cells, lymphoid cells, macrophages, neutrophils, neurons, and mast cells (Figure 3a,b). Patients with HF exhibited increased ratios of fibroblasts, lymphoid cells, smooth muscle cells, and macrophages. Conversely, the ratios of endothelial cells and neutrophils were decreased (Figure S4A). We used the “GSVA” package to evaluate the lactylation enrichment scores of each cell in the scRNA-seq dataset [17]. As shown in Figure S4B, fibroblasts, endothelial_cells, smooth_muscle_cells, neurons, and mast cells displayed relatively high lactylation level scores, whereas macrophages exhibited relatively low lactylation level scores. To further investigate the key pathways associated with lactylation, each cell was scored based on the dataset from MSigDB using the R package GSVA [29]. GO analysis indicated that these genes are mainly enriched in the processes of 5-phosphoribose 1-diphosphate metabolic process and 3’ UTR- mediated mRNA destabilization (Figure S4C). HALLMARK pathway scores were analyzed across different cell types, and the “angiogenesis”, “apoptosis”, “androgen response”, and “epithelial–mesenchymal transition” pathways showed the strongest correlations across all cell types (Figure S4D). KEGG pathway enrichment analysis revealed that the hub lactylation-related genes were primarily involved in pathways such as ABC transporters, arachidonic acid metabolism, and antigen processing and presentation pathways (Figure S5E). Of note, the enrichment analysis of biological pathways was obtained by in silico inference. These predicted pathways are hypothesis-generating and require further wet-lab experiments to validate the underlying mechanisms. As shown in Figure 3c–g, *PPM1G* was predominantly expressed in fibroblasts, *EIF4G1* was predominantly expressed in endothelial cells, *HMGN2* was predominantly expressed in smooth muscle cells, and *LSP1* was predominantly expressed in lymphoid cells.

### 3.5. External Validation of Hub LRGs Expression and Diagnostic Value in Patients with HFrEF

**Figure 4:**
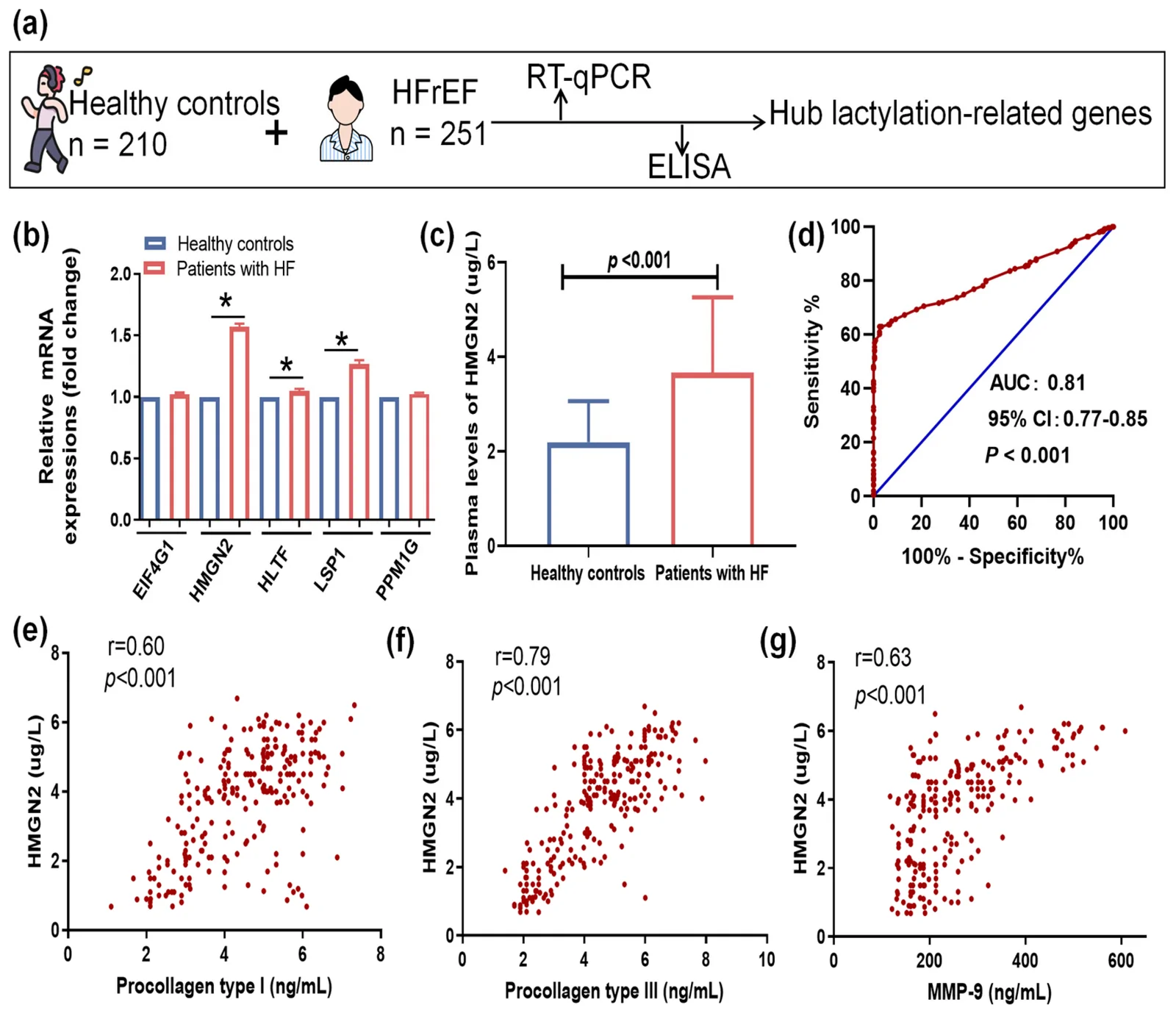
Validation of hub lactate–related genes in an independent cohort. (**a**) Flowchart for screening of hub lactylation–related genes. (**b**) The mRNA expression of five hub lactylation–related genes. (**c**) Serum concentrations of HMGN2. (**d**) Receiver operating characteristic curve showing the predictive accuracy of serum HMGN2 levels for identifying heart failure. (**e**–**g**) The relationship between serum HMGN2 levels and the fibrotic parameters. * represents *p* < 0.01.

To further confirm the findings of the integrated bioinformatics analysis, we analyzed the expression of hub LRGs in patients with HFrEF in an external validation cohort (Figure 4a). Baseline clinical characteristics were comparable between healthy controls and patients with HFrEF (Table S4). Compared with healthy controls, patients with HFrEF displayed higher heart rates, a higher incidence of coronary artery disease, and increased circulating lactate, procollagen type I, procollagen type III, MMP-9, and NT-proBNP levels. In contrast, serum HCO_3_^−^ concentrations were reduced in patients with HF. We next analyzed the mRNA expression levels of the five hub LRGs in PBMCs from patients with HF. The mRNA levels of HMGN2, LSP1, and HLTF were significantly upregulated in patients with HFrEF, whereas EIF4G1 and PPM1G showed no significant differences between patients with HFrEF and healthy controls (Figure 4b). Subsequently, we performed an enzyme-linked immunosorbent assay to assess the protein levels of HMGN2, LSP1, and HLTF in patients with HFrEF. HMGN2 levels were markedly elevated in patients with HFrEF (Figure 4c), whereas LSP1 and HLTF showed no significant differences between patients with HFrEF and healthy controls. Considering that HFrEF is characterized by severe fibrotic remodeling, we analyzed the relationship between HMGN2 levels and fibrotic parameters. HMGN2 levels in patients with HFrEF positively correlated with the levels of procollagen type I (r = 0.60; *p* < 0.001), procollagen type III (r = 0.79; *p* < 0.001), and MMP-9 (r = 0.63; *p* < 0.001) (Figure 4e–g). ROC analysis revealed excellent predictive value of serum HMGN2 levels for HFrEF (AUC = 0.81; 95% CI, 0.77–0.85; *p* < 0.001), with a cutoff value of 3.63 μg/L (Figure 4d). Logistic regression analysis showed that HMGN2 levels were an independent predictor of HFrEF (odds ratio = 1.25, 95% CI = 1.05–1.48, *p* < 0.001) (Table 1). Taken together, these results suggest that HMGN2 was significantly associated with an increased risk of HFrEF.

We added direct comparisons between HMGN2 and the established biomarker NT-proBNP in the same cohort. The AUC was 0.95 (95% CI, 0.92–0.97) for NT-proBNP. Taken together with the head-to-head and incremental discrimination analyses, our findings indicate that HMGN2 is not a substitute for NT-proBNP and remains a candidate biomarker requiring prospective validation (Details are provided in the Supplementary File).

### 3.6. Association Between HMGN2 and Phenotypic Characteristics of HFrEF

The 251 patients with HFrEF were divided into two groups based on the HMGN2 cutoff value: the L-HMGN2 group (HMGN2 < 3.63 μg/L; *n* = 93) and the H-HMGN2 group (HMGN2 ≥ 3.63 μg/L; *n* = 158). As shown in Table 2, patients in the L-HMGN2 group exhibited lower NT-proBNP levels, lactate levels, left atrial volume index (LAVI), average E/e′ ratio, IL-6, MMP-9, procollagen type I, and Procollagen type III than those in the H-HMGN2 group. Conversely, they demonstrated higher GLS values and HCO3- levels. Thus, higher HMGN2 expression was consistently accompanied by more severe inflammatory responses, fibrosis, and worse cardiac function.

### 3.7. Baseline HMGN2 Levels and Clinical Outcomes

**Figure 5:**
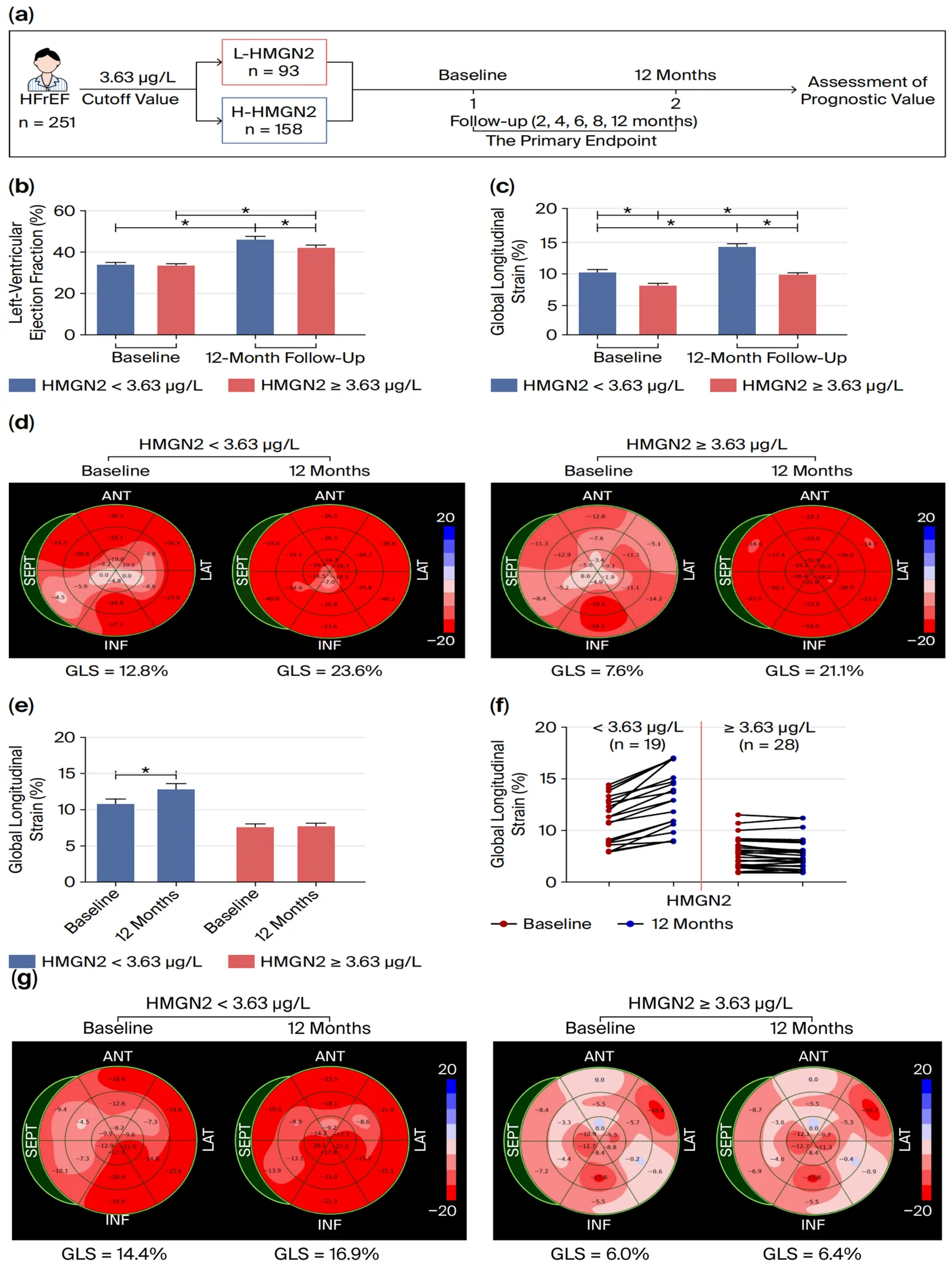
Evaluation of the association between HMGN2 and clinical outcomes in patients with heart failure with reduced ejection fraction. (**a**) Study flowchart. (**b**) Changes in left ventricular ejection fraction from baseline to month 12. (**c**,**d**) Changes in GLS from baseline to month 12. (**e**,**f**) Changes in GLS in patients without improved ejection fraction from baseline to month 12. (**g**) GLS values of a patient without improved ejection fraction from baseline to month 12. Abbreviations: GLS, global longitudinal strain. * represents *p* < 0.01.

We further evaluated the association between baseline serum HMGN2 and subsequent clinical outcomes, as well as its association with changes in cardiac function in patients with HFrEF (Figure 5a). During the 12-month follow-up period, we observed improved LVEF and GLS at 12 months in both the L-HMGN2 and H-HMGN2 groups. Patients in the H-HMGN2 group tended to achieve a greater magnitude of LVEF improvement (Figure 5b). Notably, although there was no difference in LVEF between the two groups at baseline, patients in the L-HMGN2 group exhibited higher GLS values than those in the H-HMGN2 group (Figure 5c,d). We further analyzed the GLS values in the patients without improved EF (defined as a ≤10% absolute LVEF improvement) at the 12-month follow-up. At the 12-month follow-up, EF did not improve in 19 patients from the L-HMGN2 group and 28 patients from the H-HMGN2 group. While GLS improved in 19 L-HMGN2 patients (*p* = 0.02), 28 H-HMGN2 patients showed no GLS alteration (*p* = 0.79) (Figure 5e,f). As shown in Figure 5g, one patient in the L-HMGN2 group maintained an LVEF of 37% from baseline to 12-month follow-up in a patient from the L-HMGN2 group. Notably, GLS values for the mid-interventricular septum and mid-lateral wall were significantly upregulated, and the average GLS value increased from 14.4% to 16.9% at 12-month follow-up. In contrast, another patient from the H-HMGN2 group without EF improvement showed no alteration in GLS. Considering that GLS can reflect early left ventricular systolic dysfunction and regional myocardial function, our findings suggested that patients with lower HMGN2 levels exhibit better improvement in regional myocardial systolic function. Thus, HMGN2 may serve as a sensitive biomarker for cardiac function assessment. It should be noted that the follow-up echocardiography and 6MWT analyses were limited to a subset of participants, which may introduce selection bias and restrict generalizability.

**Figure 6:**
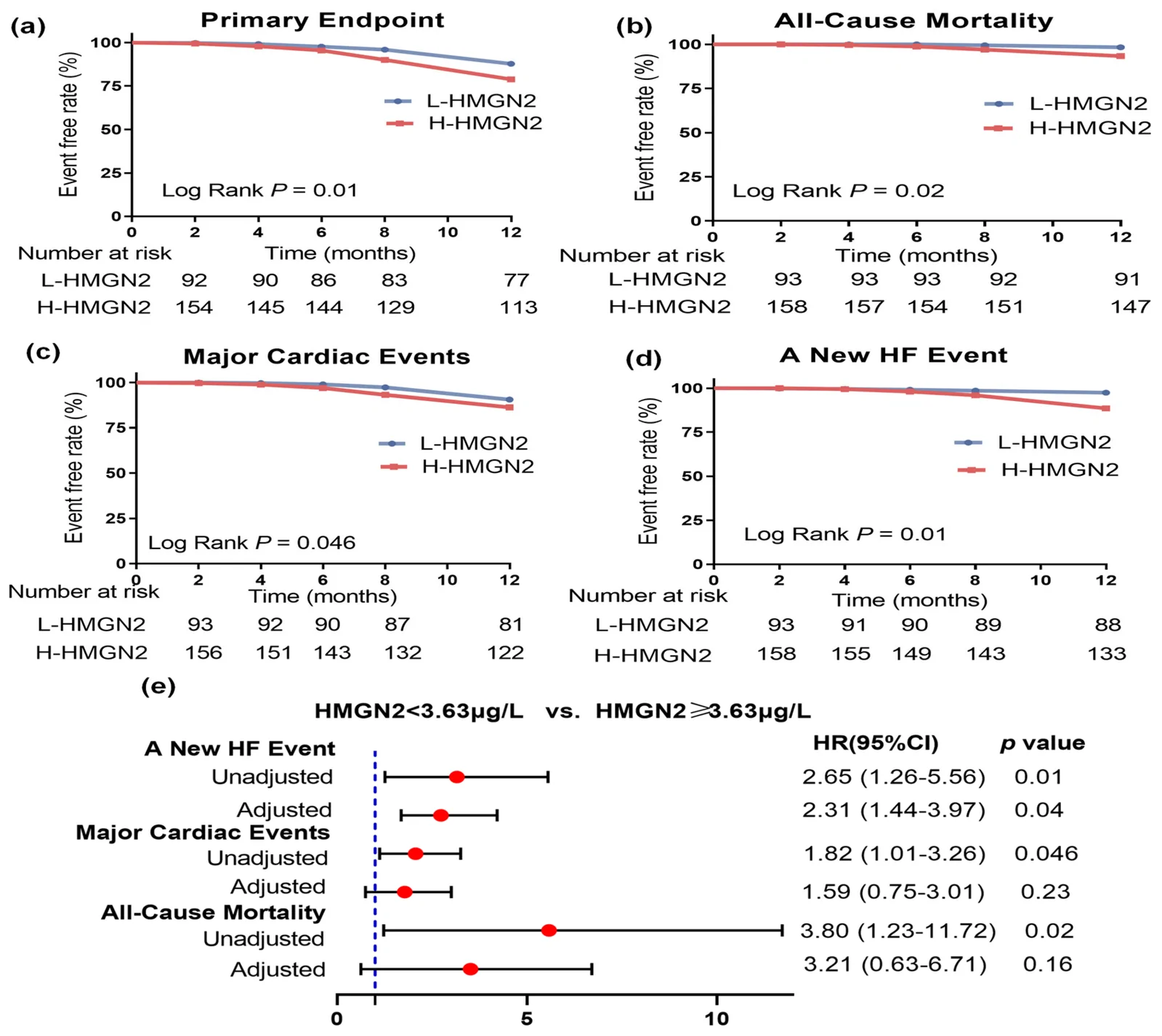
Evaluation of the association between HMGN2 and clinical outcomes in patients with heart failure with reduced ejection fraction. Survival analysis illustrating the association between the levels of HMGN2 and the primary endpoint: (**a**) Primary endpoint. (**b**) All–cause mortality. (**c**) Major cardiac events. (**d**) A new HF event. (**e**) Likelihood of major cardiac events, new HF events and all–cause mortality (higher vs. lower HMGN2 levels) after adjustment for baseline covariates.

During the 12-month follow-up period, 61 participants (24%) reached the composite primary endpoint. Among them, 48 cases of major cardiac events and 13 cases of all-cause mortality. Elevated HMGN2 levels were significantly associated with a higher incidence of major cardiac events and all-cause mortality (Figure 6a–d). Among the 48 patients who developed major cardiac events, 30 experienced a new HF event. After adjusting for baseline measurements, including sex, age, BMI, history of coronary artery disease, atrial fibrillation, diabetes mellitus, hyperlipidemia, smoking, SBP, renal dysfunction, and lactate, patients with HFrEF with elevated HMGN2 levels were associated with a higher risk of new HF events than those with lower HMGN2 levels (Figure 6e). These findings suggest that patients with high HMGN2 expression are more susceptible to new HF events. However, this observation is limited by the relatively small sample size of the present cohort; larger prospective cohorts are required to validate this result in future investigations.

**Figure 7:**
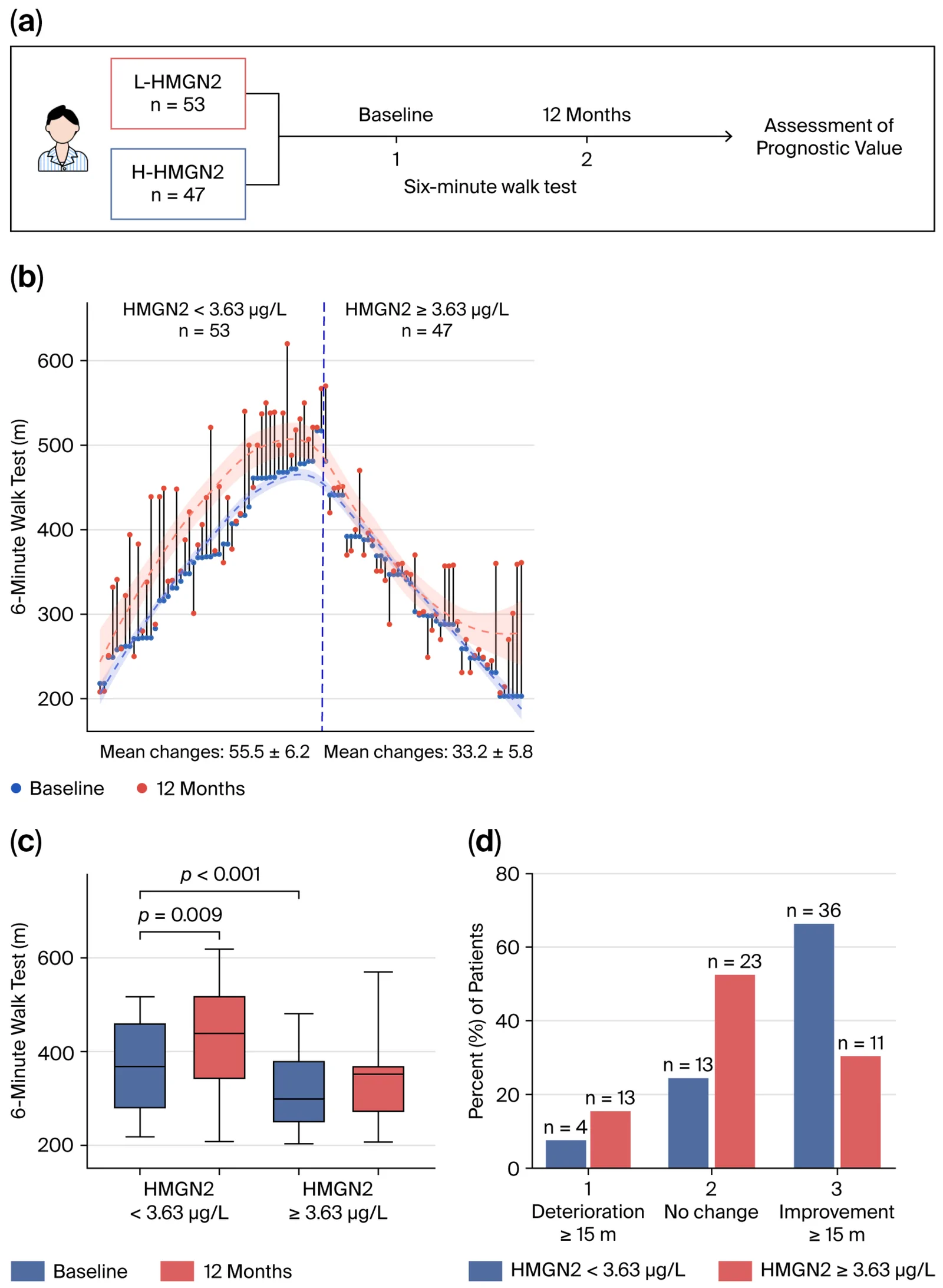
Baseline values and changes in 6-min walk test distance stratified by HMGN2 levels. (**a**) Study flowchart. (**b**,**c**) Changes in 6MWT distance in meters from baseline to month 12. (**d**) Data are represented as the proportion of patients experiencing 6-min walk test distance deterioration, no change, or improvement per treatment group.

A 6MWT was performed at baseline and 12 months in 100 patients with HFrEF, including 53 patients in the L-HMGN2 group and 47 patients in the H-HMGN2 group (Figure 7a). Patients in the L-HMGN2 group had longer baseline 6MWT distances and experienced significantly greater improvement at 12 months than those in the H-HMGN2 group (Figure 7c). Figure 7b visually demonstrates the raw data for baseline values, changes over 12 months, and final 6MWT distances measured at 12 months for each patient, stratified by HMGN2 cutoff value. After 12 months, patients with HF who improved their 6MWT distance by ≥15 m were more likely to have lower HMGN2 levels (*n* = 36; 68%) than patients without improvement in 6MWT distance (*n* = 11; 23%; Figure 7d). We also observed a higher proportion of patients with higher HMGN2 levels who experienced deterioration in walking distance (*n* = 13; 28%) than patients with lower HMGN2 levels (*n* = 4; 8%; Figure 7d).

## 4. Discussion

A growing body of evidence indicates that lactylation modification modulates myocardial metabolic function and participates in the initiation and progression of HFrEF [30,31], and that relevant lactylation biomarkers exhibit diagnostic efficacy for HFr [32,33]. Nevertheless, research regarding the prognostic implications of lactylation-related biomarkers in patients with HFrEF remains scarce. In this study, we established an analytical framework combining single-cell transcriptomic data, RNA sequencing data, and machine learning to identify the diagnostic and prognostic utility of lactylation-related biomarkers in patients with HFrEF. Our findings provide three key insights: (1) Bioinformatics analysis and machine learning identified EIF4G1, HLTF, HMGN2, LSP1, and PPM1G as the hub LRGs in patients with HF; (2) in an external validation cohort, elevated serum HMGN2 levels were significantly associated with HFrEF. Beyond its diagnostic utility in HFrEF, HMGN2 demonstrated satisfactory predictive capacity for the occurrence of the primary endpoint and the improvement of cardiac function; and (3) HMGN2 may serve as a sensitive biomarker for the assessment of early left ventricular systolic dysfunction, particularly in scenarios where LVEF alone is insufficient to capture subtle improvements in myocardial performance.

Lactate is not just a simple metabolic waste product of glycolysis [5] but also a critical node in cardiovascular homeostasis and health [31]. Lactate dysregulation induces cardiac dysfunction and contributes to HFrEF progression. During the development of HFrEF, lactate can regulate the secretion of inflammatory cytokines by cardiac fibroblasts, decreasing the levels of Fas, IL-12p40, or fractalkine, and increasing IL-13 [34]. Some clinical studies have shown that high blood lactate levels are significantly associated with high mortality rates in patients with acute HFrEF [12]. Lactate-induced changes in histone lactylation have a significant impact on cardiac energy metabolism [8,35]. Histone H3 lysine 18 lactylation acts as a pro-pathological epigenetic driver that activates hypertrophic gene programs (e.g., TGF-β2, GATA4, and PI3K/AKT/mTOR), thereby accelerating maladaptive cardiac remodeling [13,36]. Furthermore, lactylation orchestrates macrophage polarization via the JAK2/STAT2/STAT4 axis, shifting the M1/M2 balance toward chronic inflammation and interstitial fibrosis, thereby constituting a “metabolic–epigenetic–immune” triad that perpetuates HF progression [37].

HMGN2, one of the five hub genes identified in our analysis, has been independently verified as an LRG in HF in two recent studies [33,38]. Zhang et al. recognized HMGN2, together with HLTF, ARGLU1 and LSP1, as four core LRGs markedly upregulated in HF. Meanwhile, Tan et al. [33] identified HMGN2 among six core LRGs associated with dilated cardiomyopathy and illustrated that these core LRGs regulate immunometabolic remodeling [38]. Nonetheless, the two aforementioned studies only established the diagnostic value of HMGN2 in HFrEF at the mRNA level. Neither extended their analyses to the protein level nor evaluated its prognostic potential. Mechanistically, as a non-histone chromosomal protein, HMGN2 reportedly competes with histone H1 for binding to chromatin and regulates the chromatin compaction capacity of histone H1 [39]. HMGN2 has also been reported to participate in oxidative stress and actin cytoskeleton rearrangement [40]. Changes in HMGN2 expression may promote histone H1 binding to chromatin and stimulate the chromatin-condensing activity of H1. Increased histone levels enhance lactylation. HMGN2 acts as a chromatin architectural protein that competitively binds nucleosomes at active enhancers and promoters [41]. This interaction reduces p300-mediated acetylation of H3K18, H3K23, and H3K27 by 40–60% [36,41]. Nevertheless, the observed positive correlations between HMGN2 and activated T-cell subsets imply that HMGN2 may also remodel chromatin accessibility at immune-related genes, facilitating the immunometabolic remodeling underlying HFrEF progression. In the present study, we confirmed upregulated HMGN2 expression at both the mRNA and protein levels in patients with HFrEF and further conducted prognostic follow-up assessments. We found that patients with HF and low serum HMGN2 levels had a lower incidence of adverse cardiac events, reduced all-cause mortality, and superior long-term improvements in both LVEF and 6MWT distance. Collectively, these findings suggest that serum HMGN2 may serve as a candidate biomarker for risk stratification in patients with HFrEF.

This study has several limitations. First, the definition of heart failure with improved ejection fraction (HFimpEF) requires a baseline LVEF ≤ 40%, an absolute increase of ≥10 percentage points in LVEF, and a follow-up LVEF > 40% [42]. However, several enrolled patients in this study exhibited baseline LVEF > 40%. Therefore, we adopted an LVEF increase of >10% as the criterion for EF improvement. Future work will enlarge the sample size and stratify patients into heart failure with reduced ejection fraction and heart failure with mildly reduced ejection fraction subgroups to facilitate more rigorous implementation of the definition for HFimpEF. Furthermore, while we validated the clinical relevance of HMGN2 in patients with HFrEF, this investigation was a single-center study with a small sample size, underscoring the need for larger, multicenter cohorts to confirm these findings; the follow-up period is relatively short for assessing true prognostic value. Our findings reflect outcomes within a 12-month window, and longer-duration follow-up will be required to assess the sustained prognostic performance of HMGN2 over extended time. Second, the specific cell types targeted by HMGN2 were not identified, highlighting an area that warrants closer inspection. Third, the gene selection criteria may exclude biologically relevant genes with modest expression changes. Further experimental studies are required to explore those genes that were filtered out in the current screening pipeline.

## 5. Conclusions

In conclusion, HMGN2 is a lactylation-associated biomarker that is elevated in patients with HFrEF, associated with prevalent HFrEF, and shows promise for risk stratification with diagnostic discrimination. Its prognostic and predictive value remains to be confirmed in independent, adequately powered prospective cohorts.

## Figures and Tables

**Table 1:** Clinical Predictors of the Achievement of HF by Univariable and Multivariable Logistic Regression Analysis.

	Univariable		Multivariable	
Baseline Variable	OR (95% CI)	*p* Value	OR (95% CI)	*p* Value
Male, *n* (%)	1.81 (0.98–3.11)	0.06	–	
BMI	1.93 (1.07–2.88)	0.02	1.56 (0.91–2.27)	0.19
Heart rate	1.72 (0.85–3.09)	0.37	–	
History of CAD	2.55 (1.31–4.02)	<0.001	2.06 (1.25–3.72)	0.003
Atrial fibrillation	1.28 (0.78–2.01)	0.09	–	
Diabetes mellitus	1.07 (0.83–1.99)	0.18	–	
Hyperlipidemia	1.44 (0.76–2.38)	0.44	–	
Smoking	0.91 (0.69–1.92)	0.56	–	
SBP	2.88 (1.57–3.77)	<0.001	1.87 (1.38–2.53)	<0.001
Renal dysfunction	1.68 (1.21–2.85)	0.003	1.53 (1.21–2.76)	0.09
Creatinine	1.32 (1.03–2.12)	0.02	1.44 (0.97–1.92)	0.06
PCO_2_	1.37 (0.68–1.95)	0.08	–	
HCO_3_^−^	2.18 (1.27–3.01)	<0.001	1.95 (0.96–2.65)	0.16
Lactate	2.35 (1.76–3.91)	<0.001	1.66 (1.37–3.28)	<0.001
Procollagen type I	1.96 (1.23–2.81)	<0.001	1.53 (1.21–2.27)	<0.001
Procollagen type III	2.19 (1.62–3.17)	0.006	1.37 (1.15–1.98)	0.02
MMP-9	1.68 (1.16–2.39)	0.02	1.41 (1.07–2.11)	0.03
NT-proBNP	2.38 (1.29–3.73)	<0.001	2.12 (1.18–2.86)	<0.001
IL-6	1.89 (1.26–2.27)	0.007	1.36 (1.13–2.15)	0.01
HMGN2	2.61 (1.72–3.88)	<0.001	2.51 (1.57–3.16)	<0.001

For multivariable logistic regression analysis, the following factors were adopted: gender, BSA, BMI, Heart rate, History of CAD, Atrial fibrillation, Diabetes mellitusn, hyperlipidemia, smoking, SBP, Renal dysfunction, Creatinine, PCO_2_, HCO_3_^−^, Lactate, Procollagen type I, Procollagen type III, MMP-9, NT-proBNP, IL-6, and HMGN2. Abbreviations: BMI, body mass index; SBP, systolic blood pressure; CAD, coronary artery disease; NT-proBNP, N-terminal pro-B-type natriuretic peptide; PCO_2_, partial pressure of carbon dioxide; SO_2_, oxygen saturation; MMP-9, matrix metallopeptidase 9; IL, interleukin; GLS, global longitudinal strain; OR, odds ratio.

**Table 2:** Characteristics of patients with HF classified by serum HMGN levels.

	Serum HMGN2 Level		
	<3.63 μg/L	≥3.63 μg/L	*p* Value
No. participants	93	158	
Demographic characteristics			
Male, *n* (%)	51 (55)	82 (52)	0.29
BMI (kg/m^2^)	26.9 ± 3.0	27.6 ± 2.7	0.44
Heart rate (bpm)	77 ± 16	79 ± 19	0.13
History of CAD *n* (%)	44 (47)	79 (50)	0.53
Atrial fibrillation *n* (%)	13(14)	30 (19)	0.26
Diabetes mellitus *n* (%)	19 (20)	34 (22)	0.3
Hyperlipidemia *n* (%)	21 (23)	42 (27)	0.06
Smoking *n* (%)	17 (18)	33 (21)	0.08
SBP (mmHg)	129 ± 11	133 ±15	0.28
Renal dysfunction *n* (%)	26 (13)	28 (15)	0.62
Echocardiographic characteristics			
LVEF (%)	34 ± 5	33 ± 6	0.67
LAVI (mL/m^2^)	24 ± 7	33 ± 5	<0.001
Average E/e’	12.7 ± 2.9	14.9 ± 2.7	0.01
GLS (%)	10.9 ± 4.2	8.2 ± 3.0	0.005
Laboratory parameters			
Creatinine (μmol/L)	72.6 ± 12.7	74.4 ± 11.9	0.31
PCO2 (mmHg)	37.9 ± 8.8	38.6 ± 9.2	0.18
HCO3- (mmol/L)	25.1 ± 1.7	21.8 ± 2.0	0.01
Lactate (mmol/L)	1.38 ± 0.7	1.91 ± 0.8	0.02
Procollagen type I (ng/mL)	3.4 ± 1.3	5.5 ± 1.5	0.03
Procollagen type III (ng/mL)	3.1 ± 1.2	5.7 ± 1.9	0.002
MMP-9 (ng/mL)	227 ± 108	329 ± 117	0.003
NT-proBNP (pg/L)	791 (479, 1399)	1957(812, 4011)	<0.001
IL-6 (ng/mL)	12.7 ± 3.6	33.7 ± 7.1	<0.001

Abbreviations: see Table 1.

## Data Availability

The original contributions presented in the study are included in the article. Further inquiries can be directed to the corresponding authors.

## Supplementary Materials

The following supporting information can be downloaded at https://www.mdpi.com/article/10.3390/diagnostics16213665/s1, Figure S1: Differential expression of Lactylation genes; Figure S2: Identification of five hub genes of lactylation; Figure S3: The correlation between hub gene expression and KEGG pathway; Figure S4: Single cell RNA sequence analysis; Figure S5: Receiver operating characteristic curve showing the predictive accuracy of serum HMGN2 levels and NT-proBNP for identifying heart failure; Table S1: 371 Lactylation-related genes; Table S2: Primer sequences; Table S3: 41 Candidate genes; Table S4: Baseline characteristics of study patients.

## Abbreviations

The following abbreviations are used in this manuscript:

AUC	Area under the curve
BMI	Body mass index
CAD	Coronary artery disease
DEGs	Differentially expressed genes
ELISA	Enzyme-linked immunosorbent assay
GLS	Global longitudinal strain
HF	Heart failure
HFrEF	Heart failure with reduced ejection fraction
HMGN2	High-mobility group nucleosomal-binding domain 2
IL	Interleukin
LAVI	Left atrial volume index
LVEF	Left ventricular ejection fraction
LRGs	Lactylation-related genes
6MWT	6-min walk test
MMP-9	Matrix metallopeptidase 9
NT-proBNP	N-terminal pro-B-type natriuretic peptide
OR	Odds ratio
PBMC	Peripheral blood mononuclear cells
PCO2	Partial pressure of carbon dioxide
SBP	Systolic blood pressure
SO2	Oxygen saturation
