# Supplementary material for: Lactylation-Related Biomarkers in Heart Failure with Reduced Ejection Fraction: Association of Serum HMGN2 with Prevalent Heart Failure and Its Diagnostic Performance
Source: Diagnostics (Basel). 2026 Nov 5;16(21):3665. doi: 10.3390/diagnostics16213665 (PMC13763362; doi:10.3390/diagnostics16213665)
Supplement: Supplementary file 1 [file diagnostics-16-03665-s001.zip › diagnostics-4601046-supplementary.pdf]

Comparison of serum HMGN2 with NT-proBNP

Head-to-head discrimination analysis was performed in the same 461 participants. The AUC was 0.95 (95% CI, 0.92–0.97) for NT-proBNP and 0.81 (95% CI, 0.77–0.85) for HMGN2. （Figure S5）. The paired DeLong test showed that HMGN2 had significantly lower discriminative accuracy than NT-proBNP (ΔAUC = −0.137; 95% CI, −0.181 to −0.094; $P = 5.5 \times 10^{-10}$). We explicitly report this finding: HMGN2 is not a substitute for NT-proBNP.

Incremental discrimination analysis. Because a candidate marker may be useful even when it is individually inferior, we then tested whether HMGN2 adds information beyond NT-proBNP. Adding HMGN2 to a model containing $\log_{10}$-transformed NT-proBNP improved discrimination (cross-validated ΔAUC = 0.015; 95% CI, 0.002–0.028; P = 0.020), with a significant likelihood-ratio test (χ^2 = 20.28; df = 1; P < 0.001) and an IDI of 0.023 (95% CI, 0.003–0.059). HMGN2 remained independently associated with HFrEF after adjustment for NT-proBNP (OR 2.20 per μg/L; 95% CI, 1.53–3.17). All these statistics were computed from out-of-fold predicted probabilities obtained by stratified five-fold cross-validation repeated 40 times, because nested models compared on in-sample predictions are artefactually favourable to the larger model. We also report a limitation of our own analysis. The continuous NRI was 0.751, but at a 0.5 risk threshold only six participants were reclassified (categorical NRI 0.007). Because the reference model already achieved an AUC of 0.95, the continuous NRI is inflated by small movements in predicted probability. We have not relied on the NRI as the principal evidence of

incremental value.

Combining the head-to-head discrimination analysis and incremental discrimination analysis, we found that HMGN2 cannot replace NT-proBNP. HMGN2 remains a candidate biomarker awaiting prospective validation.

FIGURE S1 Differential expression of Lactylation genes; A - Analysis of variance volcano plots; B - construct the protein-protein interaction network and screen hub genes; C - KEGG functional clustering analysis; D - GO functional clustering analysis

FIGURE S2 Identification of five hub genes of lactylation; A - plot for λ selection in LASSO regression; B - LASSO regression screening to obtain 27 genes; C - ranking of variable importance screened by the random forest algorithm; D - genes screened by SVM-recursive feature elimination

FIGURE S3 The correlation between hub gene expression and KEGG pathway; The GSEA analysis of KEGG based on the correlation analysis result of A - EIF4G1; B – HLTF; C - HMGN2; D - LSP1; E - PPM1G

FIGURE S4 Single cell RNA sequence analysis; A - number and percentage of cells of each cell type between healthy controls and patients with heart failure; B - different expression levels of hub lactylation -related genes in each cell type between healthy controls and patients with heart failure; C - heatmap analysis of GO enrichment for key pathways associated with hub lactylation-related genes in each cell type; D - heatmap analysis of Hallmark enrichment for key pathways associated with hub lactylation-related genes in each cell type; E - Heatmap analysis of KEGG enrichment

for key pathways associated with hub lactylation-related genes in each cell type

FIGURE S5 Receiver operating characteristic curve showing the predictive accuracy of serum HMGN2 levels and NT-proBNP for identifying heart failure

Table S1 371 Lactylation-related genes
ACAT2 LEMD3 H4C1 SMAP DDX5 PPP1CC SH3GL1 SIRT1 ACIN1 LGALS1 HCFC1 SMARCA5 DECR1 PPP1R2B DDX3X SIRT2 ADAR LRPPRC HDAC1 SMARCC1 DFFA PRAM1 DDX41 SIRT3 ADNP LSP1 HDAC2 SMARCC2 DHRS7 PRCC DDX42 USF2 AHNAK MAGOH HDGF SMC3 DHX16 PRDX1 DDX46 GHRHR ALB MAGOHB HDGFL2 SNRPA1 DHX9 PRKDC PPIA CDO1 ALDH1A1 MAP2K4 HEXIM1 SOD1 EAF1 PRPF6 PPIL4 CSN3 ALDOA MAPRE1 HIST1H1C SPR ECHDC1 PSMA7 PPM1G SOCS2 ALDOB MBD2 HIST1H2BB SPR14 EDF1 PSMC1 PPP1CB GJA1 ALYREF MBP HIST1H2BD SRP14 EEF1A1 PSME3IP1 KRT10 UPRT ARGLU1 MDC1 HIST1H2BH SRRM1 EEF1G PTBP1 LAP3 STAT5B ARID1A MKI67 HIST1H2BK SRRM2 EEF2 PTMA LCP1 MED1 ARID3A MNDA HIST1H2BL SSB EHMT2 RACGAP1 LDHB SLC29A1 ARID3B MPHOSPH6 HIST1H2BN STMN1 EIF3D RALYL ZNFX1 ERBB4 ARPP19 MSN HIST1H2BO SUB1 EIF3J RAN ZRANB2 NCOR2 BCLAF1 MTA1 HIST1H3A SUMO2 EIF4G1 RANBP2 ZYX OXTR BOLA2 MTA2 HIST1H4A TCOF1 EIF4G2 RB1 HDAC3 APLN BRD4 MYH13 HIST2H2BE TERF2 EIF4H RBM10 DDX21 XDH BTF3 NCDN HIST2H2BF THOC2 EMG1 RBM14 DDX39A MT-CO2 BZW1 NCL HISTIH2BH THRAP3 ENO1 RBM17 DDX39B DDR1 BZW2 NEFL HLTF THUMPD1 ENSA RBM25 PHF6 OAS2 C19orf53 NHLRC2 HMGA1 TKT FABP5 RBM39 PKM2 VEGFA CACYBP NOC3L HMGB1 TMA7 FAM50A RBMX POLDIP3 HK2 CALD1 NOLC1 HMGN1 TMPO FKBP3 RCC2 KHSRP NME1 CALM1 NONO HMGN2 TMSB4X FLYWCH2 RECQL KIF2C CSN2 CALML5 NOP2 HMGN3 TOP2B FUBP1 RFC1 KRT1 STAT5A CALR NPM1 HMGN4 TP53 G6PD RFC4 ZNF207 ATP7A CBR1 NSUN2 HNRNPA1 TPM4 GAPDH RIMS1 ZNF280C ATP7B CBX3 NUCKS1 HNRNPC TPR GATAD2A RPA1 ZNF706 MTX1 CBX5 NUDT21 HNRNPD TRIM28 GATAD2B RPL13 H3C1 FOXB1 CCNA2 NUDT5 HNRNPF TRIR GFAP RPL14 H3C15 XBP1 CCT5 NUP133 HNRNPH1 TSSC4 GIGYF2 RPL22 SFPQ GPAT4 CD2BP2 NUP50 HNRNPK U2AF2 GTF2F1 RPL24 H2BU1 ZBTB7B CDC5L PABPC1 HNRNPL U2SURP GTF2I RPL29 H3-3A CAV1 CDV3 PABPN1 HNRNPM UBE2E1 H1-2 RPL5 SF3A1 CCND1 CDYL PAK2 HNRNPU UBE2M H1-3 RPS11 SF3B1 PRLR CEBPZ PARP1 HSDL2 UPF1 H1-5 RPS23 ZC3H4 PRL CFDP1 PCBP1 HSPE1 VARS H2AFV RPS27A ZMYM3 VDR CHD4 PCBP2 IARS2 VIM H2AFZ RRP1B JPT2 UMPS CHERP PCMT1 IFI16 WAS H2AJ RSL1D1 KHDRBS1 NEURL1 CNN2 PCNP IK WBP11 H2AX S100A11 PGK1 CAD CNN3 PDAP1 IKZF1 WDR33 H2AZ1 S100A4 PHC3 HIF1A COPS4 PDLIM1 ILF2 WIZ H2AZ2 S100A6 DDX17 CREB1 CRABP2 PES1 ILF3 XPO5 H2BC13 SAFB DDX18 NCOA1 CSRP1 PFKM IRF2BP2 XRCC4 H2BC14 SARNP SET SLC6A3 CSRP2 PFKP JMJD1C YLPM1 H2BC18 SATB1 H2BC5 ZC3H18 CWC15 PFN1 JPT1

Table S2 Primer sequences

Primer	Forward (5'-3')	Reverse (5'-3')
Actin β	CCCATGCCATCCTGCGTCTG	GCCGTGGTGGTGAAGCTGTAG
EIF4G1	TGTGGATGATGGTGGCTGGAAC	TTGGTGATCTTGGTGAGTCGTGAG
HLTF	CCACGCCTCTCATATCCAACTTTC	CGTAGTCCAACCACATGACCTCTC
HMGN2	AGAAAGGCTGAAGGGGATGCTAAG	TTAGGCTTGGGCTCTGGCTTTG
LSP1	CACCTGGAGGAGTTGAGTCTGAG	TCCTGGGCTGCTGACATTTCTG
PPM1G	GGAGGCTGAAGAGGACGATGAAG	ACCACCGCTGTTGTACCACTG

Table S3 41 Candidate genes

ALDH1A1, ARGLU1, DDX21, EIF4G1, ENO1, FAM50A, G6PD, GATAD2A, H2AFZ, HIST1H1C, HLTF, HMGN1, HMGN2, HMGN3, LCP1, LSP1, MBD2, MBP, NOP2, NUDT5, PFKM, PPM1G, PPP1CC, PTBP1, S100A11, SH3GL1, TKT, TPR, VARS, WIZ, XRCC4, ZYX, CDO1, SOCS2, GJA1, OAS2, HK2, NME1, ZBTB7B, CCND1, HIF1A

Table S4 Baseline characteristics of study patients

	Control	HF	*P* value
No.participants	210	253	
Male, n (%)	107 (51)	133 (53)	0.73
BMI (kg/m^2)	27.6 (2.9)	27.3 (3.1)	0.61
Heart rate (bpm)	71 (19)	79 (16)	0.003
History of CAD n (%)	139 (66)	123（49)	<0.001
Hypertension n (%)	105 （50)	120（48)	0.09
Atrial fibrillation n (%)	32 (15)	43（17)	0.29
Diabetes mellitusn n (%)	42 (20)	53 （21)	0.35
Hyperlipidemian n (%)	57 （27)	63 （25)	0.11
Smokingn n (%)	44 (21)	50 （20)	0.23
SBP (mmHg)	126 (12)	131 (13)	0.16
Renal dysfunction n (%)	26 (12)	28 (11)	0.56
Creatinine (μ mol/L)	71.8 (12.7)	73.1 (13.3)	0.23
PCO2 (mmHg)	37.7 (9.1)	38.1 (8.9)	0.31
HCO3$^-$ (mmol/L)	26.7 (2.1)	23.5 (1.8)	0.01
Lactate (mmol/L)	0.88 (0.6)	1.73 (0.8)	<0.001
Procollagen type I (ng/mL)	3.1 (1.0)	4.4 (1.3)	0.04
Procollagen type Ⅲ (ng/mL)	2.9 (1.1)	4.4 (1.5)	0.02
MMP-9 (ng/mL)	201 (112)	299 (105)	0.01
NT-proBNP (pg/L)	23 (15, 29)	1757 (686, 4064)	<0.001

Abbreviation: BMI, body mass index; SBP, systolic blood pressure; CAD, coronary artery disease; NT-proBNP: N-terminal pro-B-type natriuretic peptide; PCO2, partial pressure of carbon dioxide; SO2, oxygen saturation; MMP-9，matrix metallopeptidase 9

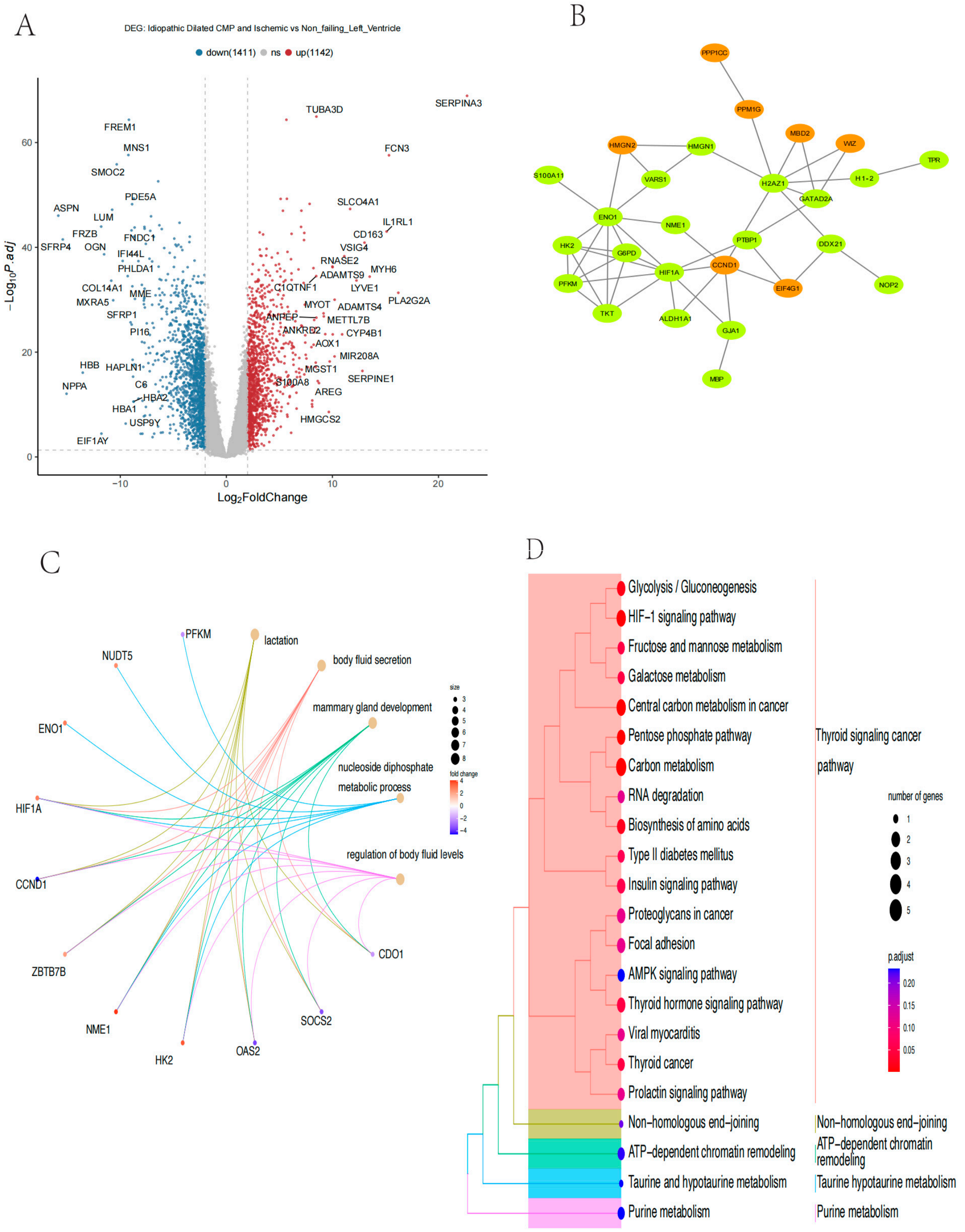
A
DEG: Idiopathic Dilated CMP and Ischemic vs Non_failing_Left_Ventricle
down(1411) ns up(1142)
SERPINA3
TUBA3D
FREM1
MNS1
FCN3
SMOC2
PDE5A
SLCO4A1
ASPN
LUM
IL1RL1
FRZB
FNDC1
CD163
SFRP4
OGN
VSIG4
IFI44L
RNASE2
PHLDA1
MYH6
ADAMTS9
COL14A1
C1QTNF1
LYVE1
MME
MXRA5
MYOT
PLA2G2A
ADAMTS4
SFRP1
ANPEP
METTL7B
PI16
ANKRD2
CYP4B1
AOX1
MIR208A
HBB
HAPLN1
MGST1
SERPINE1
NPPA
C6
S100A8
AREG
HBA2
HBA1
USP9Y
HMGCS2
EIF1AY
$-Log_{10}P.adj$
$Log_2FoldChange$
B
PPP1CC
PPM1G
MBD2
HMGN2
HMGN1
WIZ
TPR
S100A11
VARS1
H2AZ1
H1-2
GATAD2A
ENO1
NME1
PTBP1
DDX21
HK2
G6PD
HIF1A
CCND1
PFKM
EIF4G1
NOP2
TKT
ALDH1A1
GJA1
MBP
C
PFKM
lactation
NUDT5
body fluid secretion
mammary gland development
ENO1
nucleoside diphosphate metabolic process
HIF1A
regulation of body fluid levels
CCND1
CDO1
ZBTB7B
SOCS2
NME1
OAS2
HK2
size
fold change
D
Glycolysis / Gluconeogenesis
HIF-1 signaling pathway
Fructose and mannose metabolism
Galactose metabolism
Central carbon metabolism in cancer
Pentose phosphate pathway
Carbon metabolism
RNA degradation
Biosynthesis of amino acids
Type II diabetes mellitus
Insulin signaling pathway
Proteoglycans in cancer
Focal adhesion
AMPK signaling pathway
Thyroid hormone signaling pathway
Viral myocarditis
Thyroid cancer
Prolactin signaling pathway
Non-homologous end-joining
ATP-dependent chromatin remodeling
Taurine and hypotaurine metabolism
Purine metabolism
Thyroid signaling cancer pathway
number of genes
p.adjust
Non-homologous end-joining
ATP-dependent chromatin remodeling
Taurine hypotaurine metabolism
Purine metabolism

Figure S1

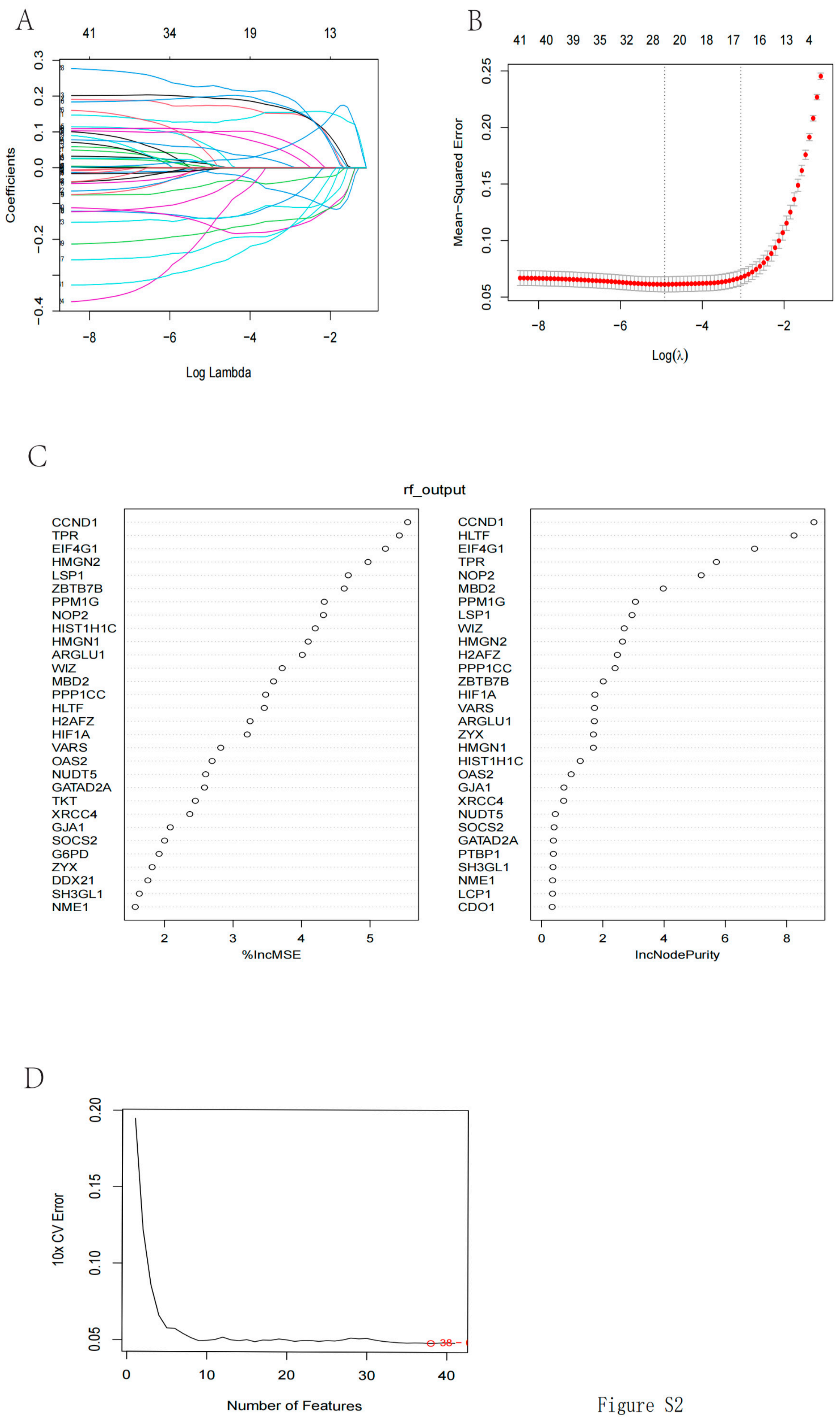

A
41 34 19 13
Coefficients
Log Lambda
B
41 40 39 35 32 28 20 18 17 16 13 4
Mean−Squared Error
Log(λ)
C
rf_output
CCND1
TPR
EIF4G1
HMGN2
LSP1
ZBTB7B
PPM1G
NOP2
HIST1H1C
HMGN1
ARGLU1
WIZ
MBD2
PPP1CC
HLTF
H2AFZ
HIF1A
VARS
OAS2
NUDT5
GATAD2A
TKT
XRCC4
GJA1
SOCS2
G6PD
ZYX
DDX21
SH3GL1
NME1
%IncMSE
CCND1
HLTF
EIF4G1
TPR
NOP2
MBD2
PPM1G
LSP1
WIZ
HMGN2
H2AFZ
PPP1CC
ZBTB7B
HIF1A
VARS
ARGLU1
ZYX
HMGN1
HIST1H1C
OAS2
GJA1
XRCC4
NUDT5
SOCS2
GATAD2A
PTBP1
SH3GL1
NME1
LCP1
CDO1
IncNodePurity
D
10x CV Error
Number of Features

Figure S2

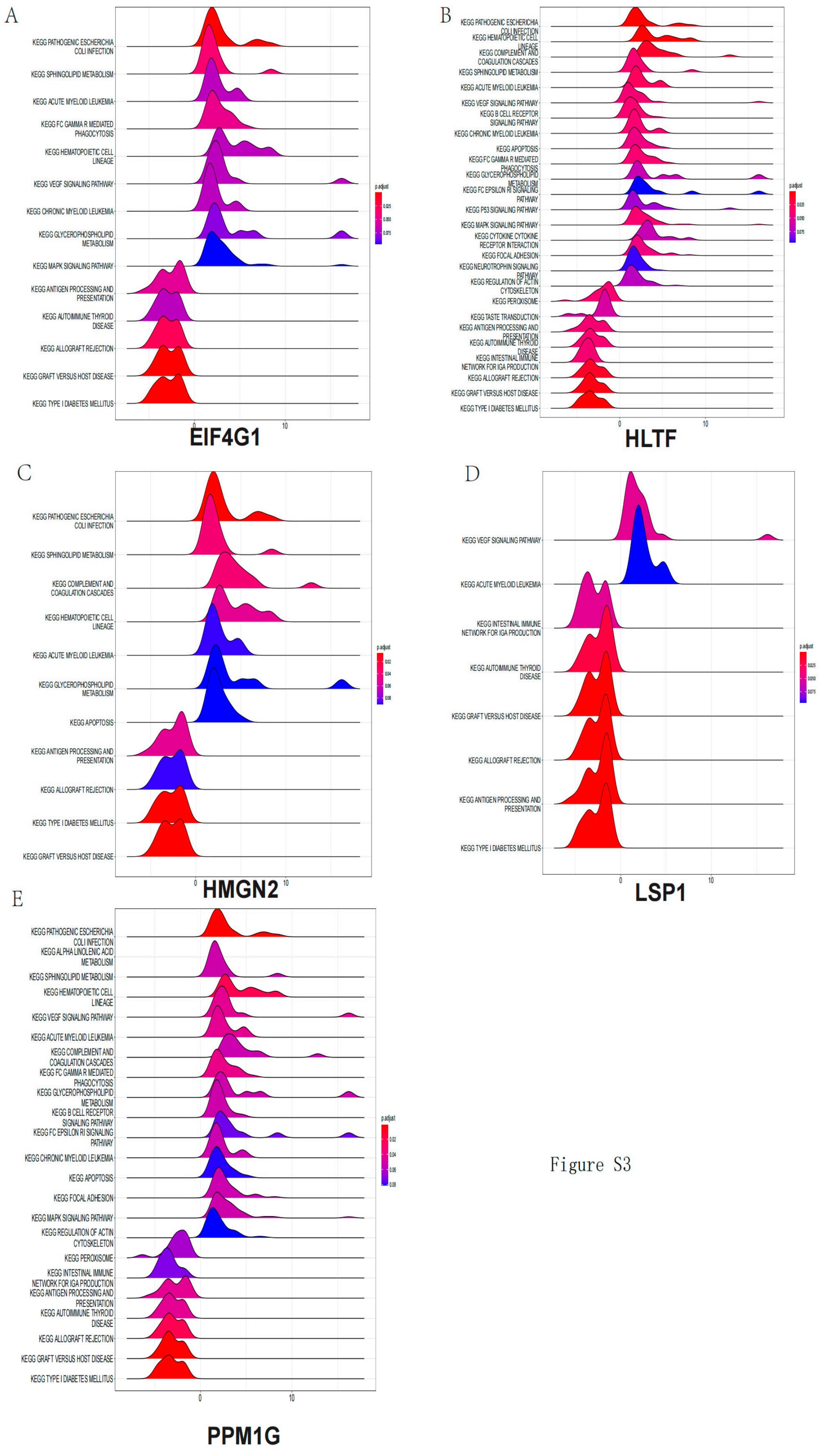

Figure S3

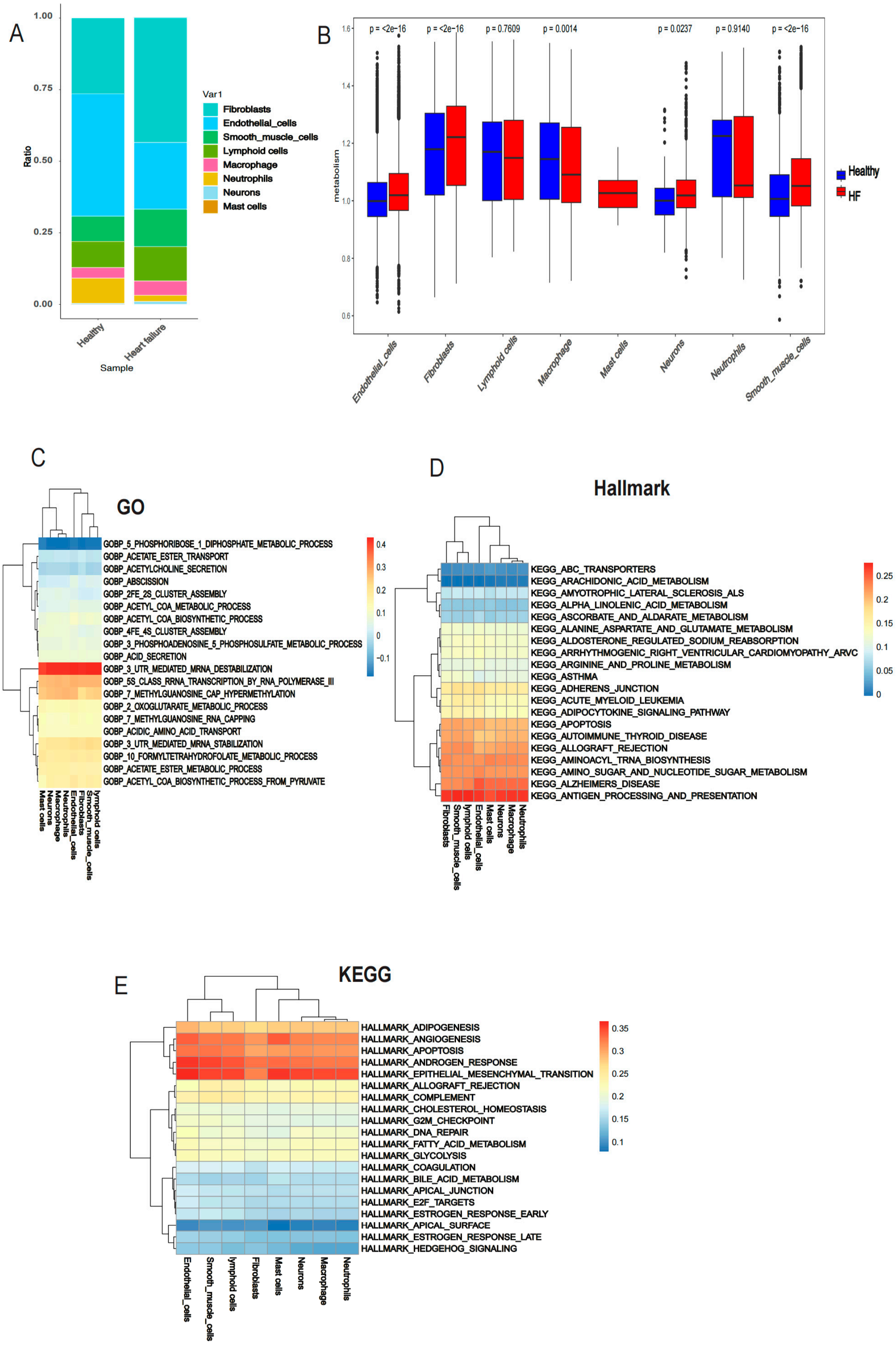

A
1.00
0.75
0.50
0.25
0.00
Ratio
Healthy
Heart failure
Sample
Var1
Fibroblasts
Endothelial_cells
Smooth_muscle_cells
Lymphoid cells
Macrophage
Neutrophils
Neurons
Mast cells
B
p = <2e-16
p = <2e-16
p = 0.7609
p = 0.0014
p = 0.0237
p = 0.9140
p = <2e-16
metabolism
1.6
1.4
1.2
1.0
0.8
0.6
Healthy
HF
Endothelial_cells
Fibroblasts
Lymphoid cells
Macrophage
Mast cells
Neurons
Neutrophils
Smooth_muscle_cells
C
GO
GOBP_5_PHOSPHORIBOSE_1_DIPHOSPHATE_METABOLIC_PROCESS
GOBP_ACETATE_ESTER_TRANSPORT
GOBP_ACETYLCHOLINE_SECRETION
GOBP_ABSCISSION
GOBP_2FE_2S_CLUSTER_ASSEMBLY
GOBP_ACETYL_COA_METABOLIC_PROCESS
GOBP_ACETYL_COA_BIOSYNTHETIC_PROCESS
GOBP_4FE_4S_CLUSTER_ASSEMBLY
GOBP_3_PHOSPHOADENOSINE_5_PHOSPHOSULFATE_METABOLIC_PROCESS
GOBP_ACID_SECRETION
GOBP_3_UTR_MEDIATED_MRNA_DESTABILIZATION
GOBP_5S_CLASS_RRNA_TRANSCRIPTION_BY_RNA_POLYMERASE_III
GOBP_7_METHYLGUANOSINE_CAP_HYPERMETHYLATION
GOBP_2_OXOGLUTARATE_METABOLIC_PROCESS
GOBP_7_METHYLGUANOSINE_RNA_CAPPING
GOBP_ACIDIC_AMINO_ACID_TRANSPORT
GOBP_3_UTR_MEDIATED_MRNA_STABILIZATION
GOBP_10_FORMYLTETRAHYDROFOLATE_METABOLIC_PROCESS
GOBP_ACETATE_ESTER_METABOLIC_PROCESS
GOBP_ACETYL_COA_BIOSYNTHETIC_PROCESS_FROM_PYRUVATE
0.4
0.3
0.2
0.1
0
-0.1
Mast cells
Neurons
Macrophage
Neutrophils
Endothelial_cells
Fibroblasts
Smooth_muscle_cells
lymphoid cells
D
Hallmark
KEGG_ABC_TRANSPORTERS
KEGG_ARACHIDONIC_ACID_METABOLISM
KEGG_AMYOTROPHIC_LATERAL_SCLEROSIS_ALS
KEGG_ALPHA_LINOLENIC_ACID_METABOLISM
KEGG_ASCORBATE_AND_ALDARATE_METABOLISM
KEGG_ALANINE_ASPARTATE_AND_GLUTAMATE_METABOLISM
KEGG_ALDOSTERONE_REGULATED_SODIUM_REABSORPTION
KEGG_ARRHYTHMOGENIC_RIGHT_VENTRICULAR_CARDIOMYOPATHY_ARVC
KEGG_ARGININE_AND_PROLINE_METABOLISM
KEGG_ASTHMA
KEGG_ADHERENS_JUNCTION
KEGG_ACUTE_MYELOID_LEUKEMIA
KEGG_ADIPOCYTOKINE_SIGNALING_PATHWAY
KEGG_APOPTOSIS
KEGG_AUTOIMMUNE_THYROID_DISEASE
KEGG_ALLOGRAFT_REJECTION
KEGG_AMINOACYL_TRNA_BIOSYNTHESIS
KEGG_AMINO_SUGAR_AND_NUCLEOTIDE_SUGAR_METABOLISM
KEGG_ALZHEIMERS_DISEASE
KEGG_ANTIGEN_PROCESSING_AND_PRESENTATION
0.25
0.2
0.15
0.1
0.05
0
Fibroblasts
Smooth_muscle_cells
lymphoid cells
Endothelial_cells
Mast cells
Neurons
Macrophage
Neutrophils
E
KEGG
HALLMARK_ADIPOGENESIS
HALLMARK_ANGIOGENESIS
HALLMARK_APOPTOSIS
HALLMARK_ANDROGEN_RESPONSE
HALLMARK_EPITHELIAL_MESENCHYMAL_TRANSITION
HALLMARK_ALLOGRAFT_REJECTION
HALLMARK_COMPLEMENT
HALLMARK_CHOLESTEROL_HOMEOSTASIS
HALLMARK_G2M_CHECKPOINT
HALLMARK_DNA_REPAIR
HALLMARK_FATTY_ACID_METABOLISM
HALLMARK_GLYCOLYSIS
HALLMARK_COAGULATION
HALLMARK_BILE_ACID_METABOLISM
HALLMARK_APICAL_JUNCTION
HALLMARK_E2F_TARGETS
HALLMARK_ESTROGEN_RESPONSE_EARLY
HALLMARK_APICAL_SURFACE
HALLMARK_ESTROGEN_RESPONSE_LATE
HALLMARK_HEDGEHOG_SIGNALING
0.35
0.3
0.25
0.2
0.15
0.1
Endothelial_cells
Smooth_muscle_cells
lymphoid cells
Fibroblasts
Mast cells
Neurons
Macrophage
Neutrophils

Figure S4

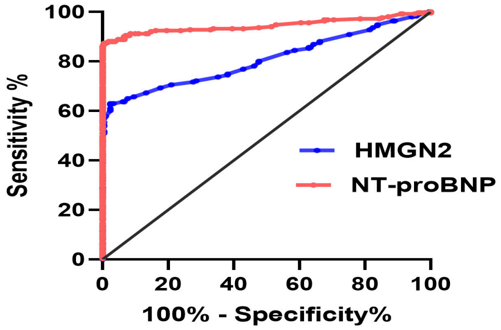

		AUC	95%CI	*P* value
	HMGN2	0.81	0.77-0.85	<0.001
	NT-proBNP	0.95	0.92-0.97	<0.001

Figure S5
